# More Meditation, Less Habituation? The Effect of Mindfulness Practice on the Acoustic Startle Reflex

**DOI:** 10.1371/journal.pone.0123512

**Published:** 2015-05-06

**Authors:** Elena Antonova, Paul Chadwick, Veena Kumari

**Affiliations:** 1 Department of Psychology, Institute of Psychiatry, Psychology and Neuroscience, King’s College London, London, United Kingdom; 2 National Institute of Health Research (NIHR) Biomedical Research Centre for Mental Health, South London and Maudsley National Health Services Trust, London, United Kingdom; Centre de Neuroscience Cognitive, FRANCE

## Abstract

**Background:**

*Mindfulness* as a mode of sustained and receptive attention promotes openness to each incoming stimulus, even if repetitive and/or aversive. Mindful attention has been shown to attenuate sensory habituation in expert meditators; however, others were not able to replicate this effect. The present study used acoustic startle reflex to investigate the effect of mindfulness practice *intensity* on sensory habituation.

**Methods:**

Auditory Startle Response (ASR) to 36 startling probes (12 trials x 3 block with 40ms inter-block intervals), was measured using electromyography (EMG) in three groups of participants (N = 12/group): meditation-naïve, moderate practice, and intensive practice.

**Results:**

Intensive practice group showed attenuated startle habituation as evidenced by significantly less habituation over the entire experiment relative to the meditation-naïve and moderate practice groups. Furthermore, there was a significant linear effect showing between-block habituation in meditation-naïve and moderate practice groups, but not in the intensive practice group. However, the Block x Group interaction between the intensive practice and the meditation-naive groups was not significant. Moderate practice group was not significantly different from the meditation-naïve in the overall measure of habituation, but showed significantly stronger habituation than both meditation-naïve and intensive practice groups in Block 1. Greater practice intensity was significantly correlated with slower overall habituation and habituation rate in Blocks 2 and 3 in the intensive, but not in the moderate, practice group.

**Conclusions:**

The study provides tentative evidence that intensive mindfulness practice attenuates acoustic startle habituation as measured by EMG, but the effect is modest. Moderate practice, on the other hand, appears to enhance habituation, suggesting the effect of mindfulness practice on startle habituation might be non-liner. Better understanding of the effect of mindful attention on startle habituation may shed new light on sensory information processing capacity of the human brain and its potential for de-automatisation of hard-wired processes.

## Introduction

*Mindfulness* requires a mode of sustained attention that is open, receptive [1], and directed towards present moment experience [2]. At the advanced stages of practice, it aims to overcome a habitual process of selection and fixation on a particular sensory, emotional or conceptual content, whilst inhibiting the processing of other sensory or mental stimuli. Everything that arises in awareness on a moment-by-moment basis is attended to non-preferentially, non-judgementally, and without conceptual elaboration [3]. This receptive and non-preferential awareness when maintained unwaveringly is referred to as ‘open presence’ [4].

An early electroencephalography (EEG) study [5] observed that highly experienced Zen practitioners (three Zen masters), for whom mindfulness would have become a default mode of information processing, do not exhibit the phenomenon of alpha blocking habituation (a decrease in response with repeated presentation of identical stimuli that is not due to sensory adaption or motor fatigue) normally observed in healthy individuals. During mindful attention, the lack of habituation would result from the ability to maintain the freshness of attention for each incoming stimuli [6].

Becker and Shapiro [7] compared lay Zen practitioners with the practitioners of Yoga and Transcendental Meditation in the US, as well as two control groups of meditation-naïve individuals, one of which was asked to attend to each click and the other was asked to ignore each click. No difference between the groups was observed. However, the sample of Zen practitioners was fairly small (n = 10) and heterogeneous in terms of practice duration (mean 7.5 years, range 3–20 years). The duration and/or intensity of practice may have an effect on de-automatisation of habituation.

Habituation has been extensively studied using the acoustic startle reflex (ASR), a contraction of the skeletal musculature in response to an intensive acoustic stimulus typically measured by electromyography (EMG) of the orbicularis oculi muscle. It is a ubiquitous, cross-species phenomenon, allowing for rigorous experimental stimulus control and automated measurement [8]. The ASR shows rapid habituation in healthy adults normally evident by the 4^th^-5^th^ presentation of the startling stimulus [9].

Levenson *et al* [10] reported a single-case study of an experienced Tibetan monk Matthieu Ricard (MR) measured on a set of physiological responses induced by unanticipated acoustic startle stimuli of 115-db 100-ms burst of white noise presented through hidden loudspeakers located behind MR’s head. The ASR was ranked by the experimenters based on facial muscle response. MR showed *decreased* initial reactivity during open presence as compared with a non-meditative state, as well as a group of control participants. Although the intensity of facial response to acoustic startle was diminished in MR during open presence, there was no observable habituation over 6 repetitions of the unanticipated startle.

The findings of this case study suggest that acoustic startle characteristics and startle habituation could be altered by long-term and/or intense mindfulness practice. However, the study used experimenters’ ratings, and although this could be a reliable method of quantifying the response by the experienced raters, the automated quantification is more practical and reliable, particularly in larger studies.

The purpose of this study was to investigate the effect of mindful attention on sensory habituation using the acoustic startle habituation paradigm and electromyography (EMG) for ASR quantification. We predicted that greater intensity of mindfulness practice would be associated with attenuated ASR habituation, whereas moderate practice would be unlikely to exert measurable effects on the hard-wired physiological mechanisms involved in sensory filtering.

## Methods

The study was approved by the King’s College London Psychiatry, Nursing and Midwifery Research Ethics Committee (reference: PNM/10/11-10). All participants provided written informed consent prior to study participation.

### Participants

Twenty seven lay mindfulness practitioners (25 males, 2 females) from the Tibetan Buddhist tradition practising Dzogchen or Mahamudra, a practice closely aligned both experientially and conceptually with mindfulness as formulated by Kabat-Zinn [11], [12], were recruited through UK Buddhist centres, retreats, and events. The inclusion criterion was at least 3 years of formal meditation practice of either Dzogchen or Mahamudra under the guidance of a teacher recognised by the Tibetan Buddhist tradition. Both female practitioners were post-menopausal and therefore were expected to have no difference in the startle reflex and its modulation as compared with males of similar age [13]. Fifteen healthy individuals (all male) with no experience of mindfulness practice either through meditation, yoga, martial arts, tai chi, or qigong were matched, on average, with meditators on age, years of education, and IQ as measured by a 2-subset version of Wechsler Abbreviated Scale of Intelligence [14]. All participants were right-handed, non-smokers, and were screened for mental illness, neurological abnormalities, head injury with the loss of consciousness, and past or current alcohol/drug abuse.

The data examination revealed poor psychophysiological data quality (>50% rejected responses) for 3 meditators and 3 meditation-naïve individuals; all data for these participants were excluded from analysis, with the final sample of 24 meditators and 12 meditation-naïve individuals.

#### Characteristics of the final sample

The studies of mindfulness effects on behaviour and cognition to date have used total hours of practice to operationalise practitioner’s expertise. Although this is a good index of meditation expertise, it does not reflect differences in the intensity of practice. This is particularly relevant in samples with a wide age-range, where similar hours of practice were accumulated over a markedly different number of years. Since intensity (and consistency) of formal practice is more likely to have an effect, if any, on de-conditioning hard-wired reflexes, we indexed the intensity of practice using the following formula:

HoP−(YoPx365)=IoP

where HoP is the total hours of practice, YoP is the total years of practice, 365 is the number of days in one year, and IoP is the intensity of practice. Hence, the IoP is the hours of practice below or above an accumulative practice hours at the rate of one hour a day over the years of practice, which we took as an index of a moderate practice. Thus, IoP above 0 indexes intensive practice; IoP equal or below 0 indexes moderate practice. Using this formula, we split the meditators into *moderate* (N = 12) and *intensive* (N = 12) practice groups. As can be seen in Table 1, the three groups, *meditation-naïve* (*MN*), *moderate practice* (*MP*), *intensive practice* (*IP*), were well-matched on age, years of education, and IQ. There was a statistically significant difference between *MP* and *IP* groups in total hours [p = .02] and practice intensity [p<.0001], with the difference in the total years of practice failing to reach significance [p = .129]. There was no significant correlation between the HoP and IoP in the *MP* group [p = .346], with a strong significant correlation in the *IP* group [r = .944, p <.0001].

**Table 1:** Demographic characteristics, IQ, and meditation history for *meditation-naïve*, *moderate* and *intensive* practice groups.

	Meditation-naïve (N = 120)	Moderate Practice (N = 12)	Intensive Practice (N = 12)	Statistics
	Mean (SD)	Mean (SD)	Mean (SD)	
**Sex (Male/Female)**	12/0	11/1	11/1	χ^2^_(2)_ = 2.18, p = .34
**Age**	40.67 (10.68)	50.17 (10.36)	48.42 (11.86)	F_(2,33)_ = 2.54, p = .10
**Years of Education**	17.92 (3.63)	17.91 (2.35)	18.42 (2.94)	F_(2,33)_ = .11, p = .90
**IQ (WASI, 2-subtest)**	127.00 (7.08)	127.17 (3.35)	125.00 (3.56)	F_(2,33)_ = .82, p = .45
**Years of practice**	n/a	25.25 (12.81)	18.00 (9.48)	t _(22)_ = 1.58, p = .13
**Hours of practice**	n/a	5856.50 (4399.92)	11326.25 (8232.45)	t _(22)_ = 2.03, p = .05*
**Intensity of practice**	n/a	-3359.69 (3371.87)	4756.25 (5626.67)	t _(22)_ = 4.29, p<.0001**

* p value is significant at the .05 level.

**p value is significant at the .01 level.

### Psychophysiological data collection

The eye blink startle response was indexed by recording electromyographic (EMG) activity of the right orbicularis oculi muscle by positioning two miniature silver/silver chloride electrodes (4 mm) filled with Dracard electrolyte paste (SLE, Croydon, UK). The ground electrode was placed on the right mastoid. Data were collected with participants sitting in a chair that promotes alert straight posture. The laboratory was moderately lit during data acquisition.

A commercial computerized human startle response monitoring system (Mark II, SR-Lab, San Diego, California) was used to deliver acoustic startle stimuli, and record and score the EMG activity. The startle system recorded EMG activity for 250 ms (sample interval 1 ms) from the onset of the pulse stimulus. The amplification gain control for EMG signal was kept constant for all participants. Recorded EMG activity was band-pass filtered, as recommended by the SR-Lab. Analogue bandpass filtering occurred before digitizing. The high-pass and low-pass cut-off frequencies were set at 100 Hz and 1 kHz, respectively. A 50-Hz notch filter was used to eliminate the 50-Hz interference. EMG data were scored off-line by the analytic software, providing measurements for latency to peak and amplitude of the startle response as used in our previous studies [15–18]. The analytic software contains a rolling average routine which smooth the rectified EMG response. Response onset was defined by a shift of 7.63 μV from the baseline value occurring within 20–120 ms from the onset of startle stimulus. The baseline value consisted of the average of the minimum and maximum values recorded during the first 18 ms. The latency to peak was defined as the latency to the point of maximal amplitude that occurred within 18–120 ms from the onset of startle stimuli.

### Startle habituation paradigm and procedures

The startle habituation paradigm consisted of 3 blocks of 12 acoustic startle trials with the average inter-stimulus interval of 15 seconds within each block (range 9–21 sec), and an inter-block interval of 40 sec. The startling stimulus was a 40-ms presentation of 115 dB (A) SPL white noise (rise time <1 ms) over 70 dB (A) continuous background white noise. The startle probes were delivered via the head-phones (TDH-39P, Maico). The session lasted approximately 15 min.

Each participant was played 2 startle probes as a demonstration of the stimuli before the start of the experimental session; the startle response data for these probes were not used for the analysis. The session began with a 4-min acclimatization period consisting of 70 dB (A) continuous white noise. Meditators were instructed to use this period to settle into mindfulness practice. The meditation-naïve individuals were instructed to remain alert and aware of their surroundings. All participants were asked to maintain a soft gaze on a point in front of them designated on the wall with a blue sticker. The sticker was placed to direct the gaze slightly above the horizon. Both Dzogchen and Mahamudra traditions use this slightly upward gaze to promote open spacious awareness. The meditators were instructed ‘to rest in open presence, neither paying particular attention to the startling noises nor ignoring them or attempting to suppress an eye blink in response to them, but rather treating them non-preferentially as any other experience that arise in the moment’. Meditation-naïve individuals were instructed ‘to remain alert and awake throughout the experiment, neither paying particular attention to the startling noises nor ignoring them or attempting to suppress an eye blink in response to them, allowing natural response to occur, and to return their awareness to the surroundings if they caught themselves mind-wandering’. We deliberately chose not to give control participants a meditation instruction in relation to the quality of attention and awareness apart from asking them to remain alert and aware, as this might introduce ambiguity and effortful processing demands which we wished to avoid.

### Startle habituation quantification

Startle habituation was quantified using individual regressions (i.e., regression performed on each individual’s amplitude data) following the procedure adopted by previous studies [19] [20] fitting the equation:

Y=a+bX

where *X* corresponds to the log-transformed startle stimulus number (trial number) and *Y* corresponds to the square root of the response amplitude for that stimulus. The startle stimulus number, *X*, is log-transformed based on the observation that habituation curves tend to resemble negative exponential functions. Startle amplitudes, *Y*, are square root transformed to reduce the variability, skewness, and heteroscedacity associated with extremely large physiological responses occurring in some individuals. The intercept, *a*, corresponds to the level of initial reactivity (i.e. the response amplitude to the first startle stimulus). The primary variable of interest is the slope *b*, which corresponds to the individual rate of habituation. Negative slope value indicates *decreased* responding over time, with larger negative values indicating *faster* and *steeper* habituation.

The habituation slopes for each of the 3 blocks of 12 trials and the overall habituation slope for 36 trials of the experiment were calculated for each participant.

### Behavioural measure

To control for the possible role of sustained attention on startle habituation, both groups were assessed using Continuous Performance Task, identical pairs version (CPT-IP) [21] with *d’prime* as the dependent variable indexing ability to sustain attention.

### Self-assessment measures

All participants completed the Mindfulness Attention Awareness Scale (MAAS) [2], a validated measure of dispositional mindfulness. Mindfulness practitioners also completed Freiburg Mindfulness Inventory (FMI)[22] designed to assess mindful attention in daily life in people who meditate. To further characterise the three groups of participants, we administered Beck Anxiety Inventory (BAI) [23], and Beck Depression Inventory-II (BDI-II) [24].

### Analyses

The group differences in initial reactivity (a square root of ASR amplitude to the first trial of the first block) and overall startle habituation (mean beta slope over 36 trials) were assessed using a one-way analysis of variance (ANOVA, p<.05). The group differences in latency to peak (in ms) and startle habituation (mean beta slopes) for Blocks 1, 2, & 3 were examined using 3 (Block) x 3 (Group) repeated-measures ANOVAs (p<.05). Lower order repeated measures ANOVAs were used to further probe significant main effects and their interactions, and where these were significant, were followed by planned group contrasts using t-tests (p<.05). The relationships between startle habituation and the self-report mindfulness measures were examined using Pearson product-moment correlations (p<.01).

## Results

### Startle response characteristics and habituation

**Fig 1:**
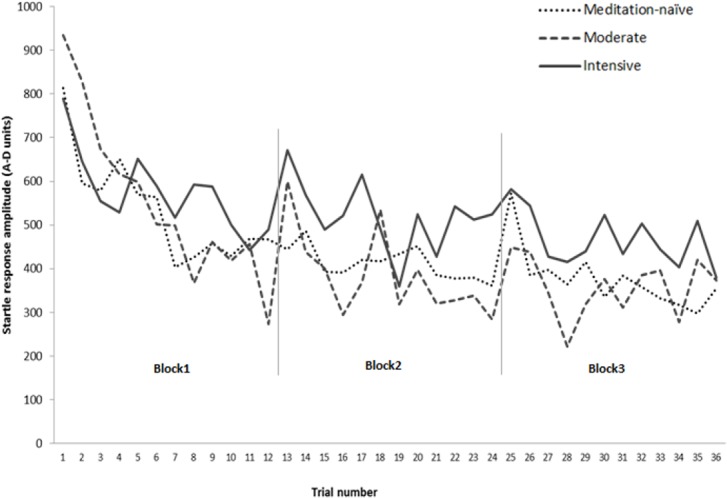
Mean startle response amplitude (A-D units) across 36 trials (3 blocks of 12 trials) for *meditation-naïve*, *moderate* and *intensive* practice groups.

Fig 1 presents the mean startle amplitude of three groups over 36 trials.

**Fig 2:**
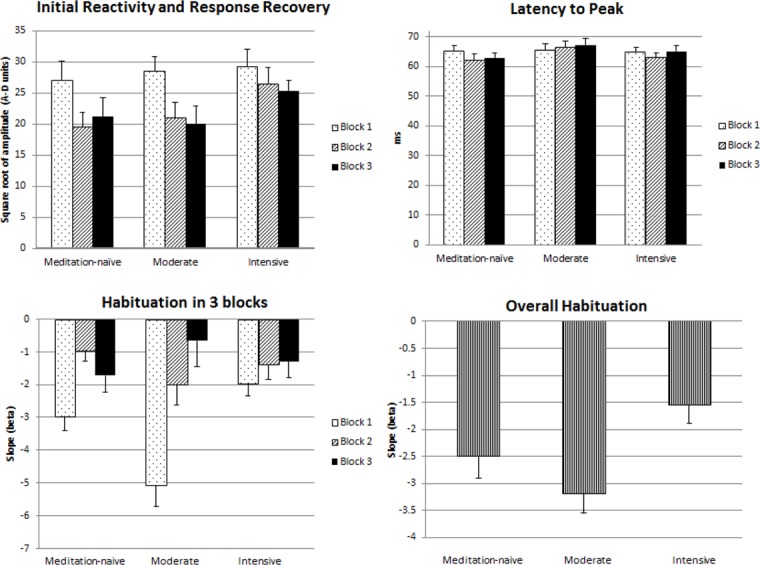
Startle response characteristics: initial reactivity (a square root of startle response amplitude to the first trial of Block 1) and response recovery (a square root of startle response amplitude to the first trial of Blocks 2 & 3); latency to peak; and startle habituation for 3 blocks and overall habituation (beta) in *meditation-naïve*, *moderate* and *intensive* practice groups.

Table 2 and Fig 2 present mean latency to peak, initial reactivity, and startle habituation for *MN*, *MP*, and *IP* groups.

**Table 2:** Mean (SD) initial reactivity (a square root of startle response amplitude to the first trial of the first block, means for blocks 2 and 3 are reported descriptively), habituation of startle response (slope), and latency to peak (ms) for *meditation-naïve*, *moderate* and *intensive* practice groups.

	Meditation-naïve (N = 12)	Moderate Practice (N = 12)	Intensive Practice (N = 12)	Repeated-Measures ANOVA
	Mean (SD)	Mean (SD)	Mean (SD)	Mean (SD)
***Initial reac*t*ivity***				
**Block 1**	27.18 (10.29)	28.50 (8.06)	29.25 (9.53)	*Block main effect*:
**Block 2**	19.59 (7.78)	21.06 (8.65)	26.52 (8.83)	F_(2,33)_ = .151, p<.861
**Block 3**	21.26 (10.57)	20.11 (9.71)	25.38 (5.55)	
***Habituation slope***				*Block main effect*:
**Block 1**	-2.96 (1.60)	-5.07 (2.25)	-1.97 (1.31)	F_(2,66)_ = 21.71, p<.0001**
**Block 2**	-0.95 (1.17)	-2.00 (2.14)	-1.39 (1.55)	*Block x group interaction*:
**Block 3**	-1.73 (1.79)	-0.67 (2.76)	-1.29 (1.71)	F_(4,66)_ = 6.05, p<.0001**
**Overall**	-2.49 (1.39)	-3.19 (1.24)	-1.39 (0.921)	One-way ANOVA:
				F_(2,33)_ = 4.98, p = .01**
***Latency to peak***				*Block main effect*:
**Block 1**	65.32 (6.00)	65.49 (7.52)	64.75 (6.15)	F_(2,66)_ = 1.20, p = .31
**Block 2**	62.31 (7.17)	66.56 (6.70)	63.07 (4.70)	*Block x group interaction*:
**Block 3**	62.94 (6.78)	67.33 (7.60)	65.06 (6.98)	F_(4,66)_ = 1.38, p = .25

** p value is significant at the. 01 level.

#### Initial reactivity

Initial reactivity did not differ [p = 0.861] between the groups [raw ASR values *MN*: Mean (SD) = 813.17 (552.36) (393.27); *MP*: 934.17 (516.52); *IP*: 788.83 (490.76)] (the mean square root of ASR amplitude to the first trial of the first block as well as blocks 2 and 3 for each group is reported in Table 1). There were no correlations between initial reactivity and the IoP in either *MP* [p = .761] or *IP* [p = .868] groups.

#### Startle habituation

Before proceeding with testing the main hypotheses, we examined whether the female participants habituation slope values were within the range of the respective group [*MP* group female: overall slope = -2.83 (range: -5.04 —.69); Block 1 slope = -4.28 (range: -8.75 —.76); Block 2 slope = -1.19 (range: -5.05 – 1.46); Block 3 slope = -.0229 (range: -3.78–6.98); *IP* group female: overall slope = -.89 (range: -2.45 -. 09); Block 1 slope = -2.81 (range: -4.09 —.08); Block 2 slope = -.13 (range: -5.15 –. 32); Block 3 slope = -.13 (range: -4.12 -. 827)]. Having confirmed that the values for female participants did not constitute outlier values that could bias the results, we did not use gender as a covariate in the analysis of variance as the validity of these tests is dependent on the variables having normal distribution, whereas gender variable is categorical and highly unbalanced within the groups (N = 1 per meditators’ group).

One-way ANOVA revealed a significant main effect of Group for the overall startle habituation slope across 36 startle trials [F_(2,33)_ = 4.98, p = .01]. Planned between-group contrasts showed that this effect was due to a significantly steeper habituation slope in *MN* [t _(22)_ = -2.41, p = .02] and *MP* groups [t _(22)_ = -3.31, p = .003] than in *IP* group, suggesting attenuated startle habituation in *IP* group across the entire experiment. *MN* vs *MP* difference was non-significant [p = .209].

To investigate whether habituation rate differed across the blocks between and within the groups, 3 (Block) x 3 (Group) repeated measures ANOVA with mean beta slopes for 3 blocks was performed and revealed a significant main effect of Block [F_(2,66)_ = 21.71, p<.0001] and a significant Block x Group interaction [F_(4,66)_ = 6.05, p<.0001]. The main effect of Block was followed up with separate repeated measures ANOVA over Block in the three groups. There were significant main effects of Block in *MN* group [F_(2,22)_ = 4.92, p = .02] and the *MP* group [F_(2,22)_ = 22.44, p<.0001], but not in *IP* group [F_(2,22)_ = 1.01, p = .381], showing non-significant habituation over the three blocks in *IP* group. Furthermore, the tests of within–subject linear contrasts were significant in *MN* [F_(1,11)_ = 4.85, p = .05] and *MP* groups [F_(1,11)_ = 29.33, p<.0001], but not in *IP* group [p = .234], further indicating gradual decrease in the ASR with repeated stimulation in *MN* and *MP* groups, but not in *IP* group.

The Block x Group interaction was investigated with 3 (Block) x 2 (Group) repeated measures ANOVAs. For *MN* vs *MP* groups comparison, the main effect of Block [F_(2,44)_ = 57.75, p<.0001] and the Block x Group interaction [F_(2,44)_ = 5.99, p = .005] were significant. Independent *t*-tests revealed significant differences in habituation for Block 1 between *MN* and *MP* groups [t_(22)_ = 2.65, p = .01], but not for Block 2 [p = .15] or Block 3 [p = .28], suggesting that Block x Group interaction is driven by *greater* habituation in Block 1 in *MP* group compared with *MN* group. For *MN* vs *IP* group comparison, the main effect of Block was significant [F_(2,44)_ = 5.35, p = .008], but the Block x Group interaction was not [p = .201]. Taken together with the results of the repeated measures ANOVAs investigating the main effect of Block reported above, this finding indicates that although *MN* group showed a significant linear decrease in ASR across the blocks and *IP* group did not, this within-block difference between two groups was not statistically significant. For *MP* vs *IP* group comparison, the main effect of Block [F_(2,44)_ = 19.57, p<.0001] and the Block x Group interaction [F_(2,44)_ = 10.67, p<.0001] were significant. Independent *t*-tests showed significant differences in habituation for Block 1 between *MP* and *IP* groups [t_(22)_ = -4.121, p<.0001], but not for Block 2 [p = .43] or Block 3 [p = .51], indicating that Block x Group interaction is due to *greater* habituation in Block 1 in *MP* group compared with *IP* group.

**Fig 3:**
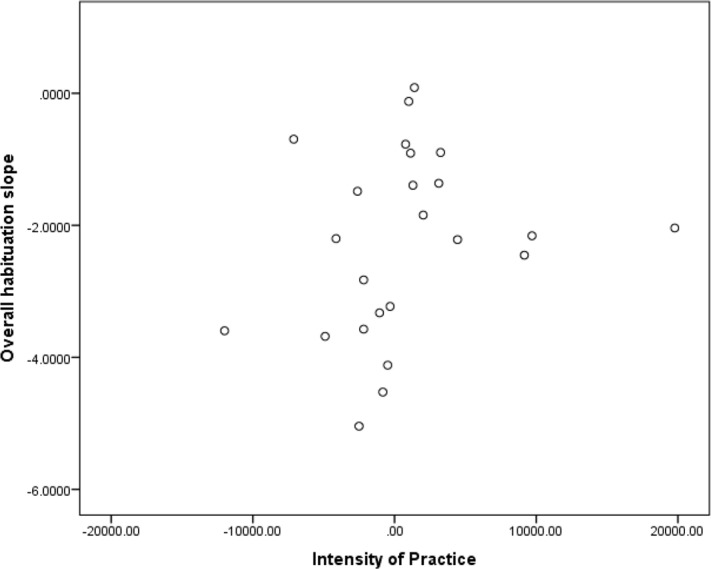
A scatterplot of Intensity of Practice and overall habituation slope for *meditation-naïve* and *intensive* practice groups MP and *IP* groups.

There were no significant correlations between the habituation slopes and the IoP in the *MP* group. In IP group, there were significant inverse correlations between the IoP and the slopes for Block 2 and 3 [Block 1: r = -.478, p = .116; Block 2: r = -.799, p = .002; Block 3: r = -.68, p = .014], and the overall slope [r = -.840, p = .001], further confirming that greater practice intensity is associated with less habituation. (See Fig 3 for the scatterplot of the overall habituation slope and the IoP values for the *MP* and *IP* groups).

#### Latency to peak

There were no significant main effects or interactions, suggesting that latency to peak did not differ between three groups in any of the blocks or within the groups across three blocks.

### Cognitive and psychological measures

Table 3 presents the means (SD) for CPT-IP and self-report questionnaires, as well as statistics for the comparison between three groups. The three groups had comparable *d*’prime of CPT-IP, anxiety (BAI), depression (BDI-II), and dispositional mindfulness (MAAS). Further in relation to mindfulness, *MP* and *IP* groups could not be differentiated using FMI.

**Table 3:** Means (SD) for CPT-IP and self-assessment questionnaires for *meditation-naïve*, *moderate* and *intensive* practice groups.

Measure	Meditation-naïve group (N = 12)	Moderate Group (N = 12)	Intensive Group (N = 12)	Statistics
	Mean (SD)	Mean (SD)	Mean (SD)	
**CPT-IP (*d*’prime)**	0.19 (.21)	0.26 (.26)	0.16 (.20)	F_(2,28)_ = 0.43, p = .66
**BAI**	3.08 (4.46)	3.33 (4.56)	1.83 (2.04)	F_(2,33)_ = 0.52, p = .60
**BDI-II**	3.18 (4.98)	1.08 (1.31)	1.50 (2.11)	F_(2,32)_ = 1.42, p = .26
**FMI**	n/a	45.08 (4.76)	45.08 (6.76)	t_(22)_ = 0.00, p = 1.00
**MAAS**	4.14 (.98)	4.65 (.36)	4.57 (.44)	F_(2,33)_ = 2.05, p = .14

**Abbreviations**: CPT-IP – Continuous Performance Task, Identical Pairs version; BAI – Beck Anxiety Inventory; BDI – Beck Depression Inventory; FMI – Freiburg Mindfulness Inventory; MAAS – Mindful Attention and Awareness Scale.

There were no correlations between startle habituation slopes and any of the self-report measures.

## Discussion

The main aim of the present study was to investigate the effect of mindfulness practice intensity on acoustic startle habituation. The results suggest that *intensive* mindfulness practice somewhat attenuates habituation to startling stimuli, whereas *moderate* mindfulness practice might enhance habituation.

Startle habituation attenuation with *intensive* practice was evidenced by significantly less habituation in *IP* group over the entire experiment vs. *MN* and *MP* groups. There was also a significant linear effect showing between-block habituation in *MN* and *MP* groups, but not in *IP* group. Furthermore, greater practice intensity was significantly correlated with slower overall habituation and habituation rate in Blocks 2 and 3 in *IP* group, but not in *MP* group. However, the habituation rate across blocks in *IP* group was not significantly different from *MN* group when testing for Block x Group interaction, suggesting that the effect, although present, was not strong enough to emerge as significant group difference in the present sample, which was relatively small.

Our finding of attenuated ASR habituation in experienced mindfulness practitioners is in line with the finding by Kasamatsu and Hirai [5] of attenuated habituation to non-startling acoustic clicks in three Zen masters using EEG. The failure to replicate the lack of habituation to acoustic clicks in lay Zen practitioners by Becker and Shapiro [7] might be due to the heterogeneity of mindfulness expertise of the studied practitioners. Our study demonstrates that it is important to take into account the intensity of practice in addition to the total hours and/or years of practice, as more intensive practice is likely to result in greater and possibly more rapid psychophysiological changes. No significant correlation between the HoP and IoP in *MP* group and a strong significant correlation in *IP* group, as well as a significant positive correlation between the IoP and the habituation slopes in *IP* but not in *MP* groups in our study further supports the utility of the IoP index in quantifying practice expertise in the absence of more objective measures and in an attempt to derive such.

The attenuated startle habituation in *IP* group could not be explained by the greater ability to sustain attention/vigilance as measured by CPT-II, as we did not observe significant group differences on the CPT-II performance. In fact, we did not observe differences in CPT-II performance in another independent sample of mindfulness practitioners compared to meditation-naïve controls in a recently published study [25], in which we report greater attentional capacity in mindfulness practitioners. MacLean *et al* [26] have reported improved performance on a CPT paradigm that used short (target) and long (non-target) vertical lines in lay practitioners after intensive training (5 hr/day for 3 months) in focused attention meditation (mindfulness of the breath) under retreat conditions as compared to wait-list controls. However, MacCoon *et al* [27] did not observe differences in sustained attention using the same version of the CPT paradigm as MacLean after MBSR as compared with active control. It is possible that findings of MacLean et al are due to more intensive practice regime that either in MacCoon’s et al or in practitioners in our study. Alternatively, the version of CPT used in the present study, which requires discriminating 4 digit numbers quickly flashing up on the screen, might be more difficult and therefore less sensitive to mindfulness as a trait.

*Intensive* mindfulness practitioners did not differ from other two groups in other ASR characteristics, including initial ASR reactivity and response latency. In the single-case study by Levenson *et al* [10] described in the introduction, MR showed *decreased* initial reactivity (ranked by the experimenters based on facial muscle response, no EMG was used) during open presence as compared with a non-meditative state, as well as a group of control participants. The lack of attenuation in the initial reactivity in *IP* group might be due to the practitioners in our study being less experienced than MR. It is also possible that the difference is due to higher intensity of startling stimuli in Levenson et al study, which produces whole-body startle, and therefore might be more sensitive in differentiating meditators from meditation-naïve individuals than the auditory startle probes used in the present study. Future research should investigate whether the initial reactivity as measured by the EMG could be prominently diminished by the long-term intensive practice. It is important to note that although the intensity of facial response was diminished in MR during open presence, there was no observable habituation over 6 repetitions of the unanticipated startle, further collaborating our finding of the effect of intensive mindfulness practice on attenuating habituation.

Together, the measures of initial reactivity and startle habituation have the potential of being developed as objective measures of mindfulness expertise. Currently, the assessment of mindfulness expertise mostly rely on self-reports. In the present study meditators with intensive meditation practice were discriminated by an objective measure (ASR habituation), but not by self-report measures of mindfulness, either dispositional (no differences in MAAS scores between three groups), or practice-related (no difference in FMI scores between two groups of meditators). These findings contribute to the existing controversy in relation to assessing trait mindfulness using self-reported measures (see [28] for the discussion of relevant issues). In demonstrating that measureable psychophysiological changes occur in the brain’s receptivity to information input following intensive mindfulness practice, the present study takes a first step towards developing startle habituation as an objective measure of mindfulness expertise. Due to the cross-sectional design of the present study it is possible, however, that the observed association between attenuated ASR habituation and practice intensity could be explained by the fact that individuals who practice intensively might display reduced ASR prior to commencing mindfulness practice and this feature of their sensory processing somehow makes the practice more appealing and thus encourages more intense practice. The correlation between practice intensity and overall habituation could in principle be explained by this direction of causality, rather than more intensive practice leading to less habituation. Future studies adopting longitudinal design should address this issue, as well as to investigate at what point in the practice expertise the quantitative and qualitative shifts in the ASR characteristics occur.

Contrary to our prediction of unchanged startle habituation with *moderate* practice, *MP* group showed significantly *stronger* habituation in Block 1 compared to both *MN* and *IP* groups. Although from Fig 1 it might appear as if this effect is due to the higher initial reactivity in *MP* group followed by normal habituation response, this is an unlikely explanation for this result. Firstly, we used square root transformed values for the initial reactivity when calculating habituation beta slope. Secondly, we explored (not reported in the results) the correlation between the square root initial reactivity values and the habituation slope for Block 1 in *MP* group. Surprisingly, the correlation was positive (r = .458), i.e. higher initial reactivity was associated with *less* habituation in *MP* group, but it was not significant (p <.135). This suggests that the initial reactivity is unlikely to explain greater habituation in Block 1 in *MP* group. It is possible that mindfulness practice might have non-linear effects on startle habituation, with moderate practice enhancing and intensive practice attenuating it. The reasons for this should be elucidated in future research provided this effect is replicable and not a chance finding in the present study.

The finding that moderate mindfulness practice enhances habituation, if confirmed by further research, has implications for clinical applications of mindfulness, particularly for the management and/or treatment of schizophrenia. Habituation is considered to be critical for efficient information processing, and if disrupted, is thought to lead to sensory inundation and cognitive fragmentation as observed in schizophrenia [8] [29]. Reduced habituation in schizophrenia patients is observed across stimulus and measurement modalities [8] [30]. Attenuated startle habituation is well-documented in schizophrenia patients, both chronic [31], [29], [9] and first-episode [32], and was shown to be stable over the course of psychotic illness in a 6-year study [33]. The limited literature on mindfulness meditation and psychosis cautions against teaching mindfulness to people with a history of [34] or vulnerable to [35] psychosis precisely due to the concern that mindfulness would promote de-automatisation of a ‘hard-wired’ process of stimuli selection and inhibition, seen as an essential condition for efficient information processing. However, mindfulness practice does not result, *per default*, in psychotic syndrome and cognitive deficits characteristic of schizophrenia. On the contrary, dispositional mindfulness [2] and mindfulness developed through training [36] are associated with reduced anxiety and stress reactivity, increased behavioural flexibility, and overall well-being. Furthermore, mindfulness training enhances performance on cognitive tasks of executive function [37], orienting attention [38], sustained attention and cognitive flexibility [39], on which schizophrenia patients exhibit performance deficits [40]. Our finding that moderate mindfulness practice was associated with enhanced startle habituation supports the use of shorter and less intensive mindfulness practice for those vulnerable to or experiencing psychosis. Indeed, Chadwick et al [41] [42] used an adapted brief 10-min mindfulness meditation in their pilot studies of the effectiveness of mindfulness for people with schizophrenia diagnoses and treatment-resistant auditory hallucinations, or paranoia, or both. The case-study [35] reported the onset of psychotic experience following an intensive meditation retreat. These findings are consistent with the proposal that it might be the intensity of meditation practice rather than meditation *per se* that could potentially induce or exacerbate psychotic states in vulnerable individuals. Equally important research question is what affords *intensive* mindfulness practitioners information receptivity whilst maintaining the integrity of information processing and even enhancing it? Future research should elucidate possible cognitive and neural mechanisms that ‘protect’ mindfulness practitioners against information overload in the presence of diminished sensory information filtering.

Since habituation is a form of non-associative learning, whereby the central nervous system automatically disregards (filters out) repetitive stimuli that hold no information value, what would be possible advantages of attenuated habituation due to mindfulness practice? Buddhist psychology posits that one of the reasons for the discontent and dis-ease that even people with no psychiatric diagnosis often experience is a rapid habituation to sensory stimulation, leading to wanting new or higher intensity experiences. Indeed, in healthy meditation-naïve adults, faster habituation is associated with impulsivity, behavioural disinhibition, and sensation seeking [43]. One of the aims of the mindfulness practice is to maintain fresh and alert attention to each incoming stimulus, no matter its valence or familiarity. This leads to experiential novelty even of the most mundane and familiar stimuli. The meditator, for example, starts to notice that every breath is somewhat different from the previous one, or that physical pain, if looked at more closely, is not a solid monolith, but a mingling ever-changing flow of sensations. To borrow from William Blake: “How do you know but ev’ry Bird that cuts the airy way, Is an immense world of delight, clos’d by your senses five?”

The main limitation of the present study is a small sample size, which might have limited the power in testing the main hypothesis and/or resulted in chance findings. The inclusion of female participants in the groups of mindfulness practitioners might have potentially affected the results, although we have confirmed that their values did not fall into the extreme ends of the range in their respective groups.

Furthermore, the observed effect of intensive mindfulness practice on attenuating startle habituation might potentially be explained by other factors not controlled for in the present study. These might include considerable changes in life style of the practitioners who practice intensively, including living in urban vs country environment, which might differentially affect central nervous system arousal overtime, choice of jobs/occupations with differential stress levels, diet, etc.

In conclusion, the present study provides preliminary and tentative evidence for attenuated startle habituation with intensive mindfulness practice on a group level and using EMG measurement. Moderate practice, on the other hand, appears to enhance startle habituation, which might have significance for understanding meditation-induced psychosis reported in previous literature and suggests that more moderate practice regimes might be advisable for people vulnerable to or suffering from psychosis. The mechanisms that protect intensive mindfulness practitioners in the face of increased information receptivity, as well as relative merits and potential ‘dangers’ of such receptivity should be explored in future research. With further research, startle habituation has the potential to be developed as an objective measure of mindfulness expertise. Further study of sensory filtering in mindfulness experts may shed new light on sensory information capacity and receptivity of the human brain and the potential for de-automatisation of hard-wired processes such as habituation.
