# Correction: More Meditation, Less Habituation? The Effect of Mindfulness Practice on the Acoustic Startle Reflex

**DOI:** 10.1371/journal.pone.0133099

**Published:** 2015-07-13

**Authors:** Elena Antonova, Paul Chadwick, Veena Kumari

There is an error in the second sentence of the “Conclusions” section of the Abstract. The correct sentence is: Moderate practice, on the other hand, appears to enhance habituation, suggesting the effect of mindfulness practice on startle habituation might be non-linear.

Throughout the fourth paragraph of the Discussion, “CPT-IP” is incorrectly referred to as “CPT-II.” The correct paragraph is: The attenuated startle habituation in *IP* group could not be explained by the greater ability to sustain attention/vigilance as measured by CPT-IP, as we did not observe significant group differences on the CPT-IP performance. In fact, we did not observe differences in CPT-IP performance in another independent sample of mindfulness practitioners compared to meditation-naïve controls in a recently published study [25], in which we report greater attentional capacity in mindfulness practitioners. MacLean *et al* [26] have reported improved performance on a CPT paradigm that used short (target) and long (non-target) vertical lines in lay practitioners after intensive training (5 hr/day for 3 months) in focused attention meditation (mindfulness of the breath) under retreat conditions as compared to wait-list controls. However, MacCoon *et al* [27] did not observe differences in sustained attention using the same version of the CPT paradigm as MacLean after MBSR as compared with active control. It is possible that findings of MacLean et al are due to more intensive practice regime that either in MacCoon’s et al or in practitioners in our study. Alternatively, the version of CPT used in the present study, which requires discriminating 4 digit numbers quickly flashing up on the screen, might be more difficult and therefore less sensitive to mindfulness as a trait.
